# Post-treatment neutrophil to lymphocyte ratio as a prognostic tool in patients treated with tocilizumab for severe COVID-19 pneumonia - a single center experience

**DOI:** 10.11613/BM.2023.020704

**Published:** 2023-06-15

**Authors:** Gomerčić Palčić Marija, Matijaca Hana, Kruljac Ivan, Vusić Lucija, Hostić Vedran, Vrbanić Luka, Fanika Mrsić, Zrilić Radovan, Ćelap Ivana, Gaćina Petar

**Affiliations:** 1 School of Medicine, University of Zagreb, Zagreb, Croatia; 2 Department of Internal Medicine, Sestre milosrdnice University Hospital Center, Division of Pulmonology, Zagreb, Croatia; 3 Department of Internal Medicine, Sestre milosrdnice University Hospital Center, Division of Hematology, Zagreb, Croatia; 4 Solmed Group, Department: Poliklinika Solmed, Zagreb, Croatia; 5 Department of Anesthesiology, Intensive Care Medicine and Pain Management, Sestre milosrdnice University Hospital Center, Zagreb, Croatia; 6 Department of Internal Medicine, Sestre milosrdnice University Hospital Center, Division of Clinical Immunology and Rheumatology, Zagreb, Croatia; 7 Polyclinic for Respiratory Diseases, Dom zdravlja Zagreb - Zapad, Zagreb, Croatia; 8 Department of Clinical Chemistry, Sestre milosrdnice University Hospital Center, Croatia; 9 School of Dental Medicine, University of Zagreb, Zagreb, Croatia

**Keywords:** tocilizumab, interleukin-6, COVID-19, neutrophils, lymphocytes

## Abstract

**Introduction:**

Tocilizumab is used in patients with severe COVID-19 pneumonia and high concentration of IL-6. We studied potential prognostic role of neutrophil and lymphocyte count regarding tocilizumab treatment.

**Materials and methods:**

We enrolled 31 patients with severe COVID-19 pneumonia and higher serum IL-6 concentration. The samples were taken on the day of tocilizumab administration and five days later. We used ROC analysis to investigate the association between the analysed parameters and 30-day mortality in order to determine the best pre-treatment and post-treatment prognostic factor. Kaplan-Meier curves and log-rank test were used to present and to analyse the difference in survival.

**Results:**

Patients had a median age of 63 (55-67) years and were treated with a median tocilizumab dose of 800 mg. During the 30-day follow-up period, 17 patients died (30-day mortality 54%). Among pre-treatment variables, neutrophil count had the best prognostic accuracy (AUC 0.81, 95%CI: 0.65-0.96, P = 0.004), while neutrophil to lymphocyte ratio (NLR) had the highest accuracy among post-treatment variables in predicting 30-day mortality (AUC 0.94, 95%CI: 0.86-1.00, P < 0.001). Among post-treatment parameters, neutrophil count and NLR were equally good prognostic factors. Post-treatment NLR cut-off of 9.8 had the sensitivity of 81% and specificity of 93%. Patients with NLR ≥ 9.8 had the median survival of 7.0 (3-10) days *vs*. median survival not reached in patients with NLR < 9.8 (P < 0.001)

**Conclusion:**

Pre-treatment and post-treatment neutrophil count with post-treatment NLR may represent prognostic tools for patients with higher IL-6 concentration in severe COVID-19 pneumonia treated with tocilizumab.

## Introduction

The coronavirus disease-19 (COVID-19) is the infection of the respiratory tract with SARS-CoV-2 virus, which is a cytopathic agent that causes lung tissue damage after intracellular infection by inducing cytolytic and thus very inflammatory kind of programmed cell death (*1*, *2*).

Hyperactivation of the immune response, including the release of pro-inflammatory cytokines such as interleukin-6 (IL-6), plays a key role in the pathophysiology of severe COVID-19 since IL-6 promotes endothelial dysfunction and the development of vascular permeability (*3*, *4*).

The accumulation of lymphocytes and inflammatory monocytes, endothelitis, apoptosis, thrombosis and angiogenesis in the pulmonary vasculature all contribute to the pathophysiology of severe COVID-19 pneumonia (*5*, *6*).

Tocilizumab is a recombinant humanized monoclonal antibody. It inhibits binding of IL-6 to its soluble and membrane receptors and is used in severe presentations of COVID-19 in order to down-modulate inflammation and to avoid respiratory and multi-organ dysfunction (*7*).

Initial studies showed that critically ill patients presented with higher concentration of inflammatory markers than the patients with low to moderate illness, while patients with lethal outcome had higher degrees of lymphopenia, neutrophilia, C-reactive protein (CRP), lactate dehydrogenase (LD), D-dimer and IL-6 production (*8*). Diverse biomarkers were used to monitor therapy with tocilizumab, but the most important response is still clinical. Severe COVID-19 pneumonia has high mortality rates and current treatment recommendations would benefit from prognostic factors regarding the use of specific therapy. Neutrophils are a heterogeneous group of cells involved in severe inflammation and thrombosis and therefore have been one of most important cells of interest during COVID-19 (*9*). Also, there is some evidence of their role in suppression of expansion of CD4+ and CD8+ T lymphocytes which then leads to lymphopenia and dysregulated anti-viral response in severe COVID-19 infection (*9*).

Results from multi-centric study by Duran-Mendez *et al.* showed that tocilizumab, a drug that blocks IL-6-CRP axis, reduces concentration of inflammatory markers such as CRP and neutrophils in severely ill patients. Its effect in reduction of inflammation, both acute and chronic, may be related to improvement of respiratory function (*10*).

Decrease in circulating neutrophils by affecting neutrophil trafficking to the bone marrow without influencing their functional capacity has been also reported in healthy subjects receiving one dose of tocilizumab (*11*). In published studies, biomarkers such as CRP, IL-6, and ferritin were proposed for selecting patients who will most likely benefit from treatment with tocilizumab but more studies are warranted to give firm conclusion. To best of our knowledge potential biomarkers for monitoring response to tocilizumab therapy are still missing. We analysed whether the neutrophil and lymphocyte count at pre-treatment and after tocilizumab treatment may be used as an additional prognostic tool in patients with severe COVID-19 pneumonia.

## Materials and methods

A prospective observational cohort study was conducted from September of 2021 to April 2022 in COVID-19 Unit, Sestre milosrdnice University Hospital Center, Zagreb, Croatia.

### Subjects

The study included 31 patients with severe COVID-19 pneumonia (described on chest computed tomography (CT)) with evident clinical deterioration, progression of hypoxemia, criteria for the syndrome of excessive cytokine secretion (fever with one or more following criteria: high concentration of CRP, IL-6 and ferritin) and concentration of IL-6 > 40 pg/mL. Those patients were treated with intravenous tocilizumab. Exclusion criteria were confirmed or probable (uncontrolled) bacterial, fungal, parasitic or other viral infection, alanine aminotransferase (ALT) and/or aspartate aminotransferase (AST) > 10 times above the normal, absolute neutrophil count < 1 x10^9^/L, platelet count < 50 x10^9^/L and pregnancy. All patients included signed informed consent and study was approved by the hospital Ethics board.

### Methods

Blood samples for routine analysis were collected repeatedly every morning in vacutainers with clot activator (biochemistry tests), K2EDTA (full blood count) and 3.2% Na-citrate (coagulation analysis) (Vaccuette, Greiner Bio-One, Kremsmünster, Austria), respectively. To obtain serum and citrate plasma, blood samples taken in tubes with clot activator and 3.2% sodium citrate were centrifuged for 10 minutes at 1800xg.

Full blood count was analysed on the Sysmex XN1000 (Sysmex Corp., Kobe, Japan), D-dimer on the BCS XP (Siemens Healthineers, Marburg, Germany), CRP and ferritin on the Abbott Architect c8000 (Abbott Laboratories, Abbott Park, USA).

In a case of suspicion of cytokine storm, additional samples in tubes with clot activator were taken for the measurement of IL-6 concentration. Serum IL-6 concentration was measured on Roche Cobas e411 (Roche Diagnostics GmbH, Mannheim, Germany).

In patients with high IL-6 concentration, tocilizumab was administered. Data was collected from the patients’ medical records through hospital information system and the laboratory results, for samples collected and analysed on the day of tocilizumab administration (pre-treatment count) and on the fifth day after tocilizumab administration (post-treatment count).

### Statistical analyses

Patient characteristics were assessed using descriptive statistics presented as median and interquartile range. Independent continuous variables were compared using Mann-Whitney test and categorical variables using the Fisher exact test. Receiver operating characteristic (ROC) analysis was performed for analysing the prognostic accuracy of each variable. Moreover, it was used to establish cut-off values for best prognostic factors and to determine their sensitivity and specificity. Kaplan-Meier curves and log-rank test were used to present and to analyse the difference in survival between groups of patients. P value of < 0.05 was considered statistically significant. The statistical analysis was performed using SPSS, Version 20 (IBM Corporation, Armonk, USA).

## Results

Our study included 24 (0.77) male and 7 (0.23) female patients with a median age of 63 (55-67) years. Patients were treated with a median tocilizumab dose of 800 (600-800) mg.

Death within 30 days occurred in 17 (0.55) patients with a median survival of 23 (7-30) days. Survivors had significantly lower pre-treatment and post-treatment leukocyte and neutrophil count and neutrophil to lymphocyte ratio (NLR), and also higher post-treatment lymphocyte count (Table 1). Pre-treatment IL-6 concentrations were not significantly different among these two groups.

**Table 1:** Comparison of the analysed parameters based on 30-day mortality

	**Non-survivors** **N = 17**	**Survivors** **N = 14**	**P**
Age (years)	63 (59-71)	60 (53-65)	0.173
Females (N, proportion)	5 (0.29)	2 (0.14)	0.412
Tocilizumab dose (mg)	800 (700-800)	680 (600-800)	0.246
Pre-treatment IL-6 (pg/mL)	143 (61-205)	65 (31-197)	0.213
Pre-treatment D-dimers (mg/L)	4.15 (1.31-4.58)	1.39 (0.6-2.93)	0.080
Pre-treatment ferritin (µg/L)	2052 (1573-2941)	1659 (1034-1934)	0.074
Pre-treatment CRP (mg/L)	57 (12-186)	54 (11-157)	0.953
Post-treatment CRP (mg/L)	7 (2-33)	2 (1-12)	0.101
Pre-treatment leukocytes (x10^9^/L)	13.2 (10.9-19.5)	7.5 (7.0-11.0)	0.005
Post-treatment leukocytes (x10^9^/L)	17.5 (9.8-19.2)	8.1 (6.2-9.9)	< 0.001
Pre-treatment neutrophils (x10^9^/L)	11.9 (9.0-18.2)	6.3 (5.4-9.3)	0.003
Post-treatment neutrophils (x10^9^/L)	15.4 (8.1-17.7)	5.2 (3.4-7.7)	< 0.001
Pre-treatment lymphocytes (x10^9^/L)	0.6 (0.5-0.9)	0.7 (0.4-1.1)	0.399
Post-treatment lymphocytes (x10^9^/L)	0.7 (0.5-1.0)	1.6 (1.0-2.2)	0.001
Pre-treatment NLR	18.6 (13.3-26.6)	8.4 (5.2-17.7)	0.009
Post-treatment NLR	22.1 (11.7-34.9)	2.7 (1.4-6.8)	< 0.001
Continuous variables are presented as median and interquartile range. IL-6 – interleukin 6. CRP – C reactive protein. NLR – neutrophil to lymphocyte ratio. P < 0.05 was considered statistically significant.			

**Figure 1:**
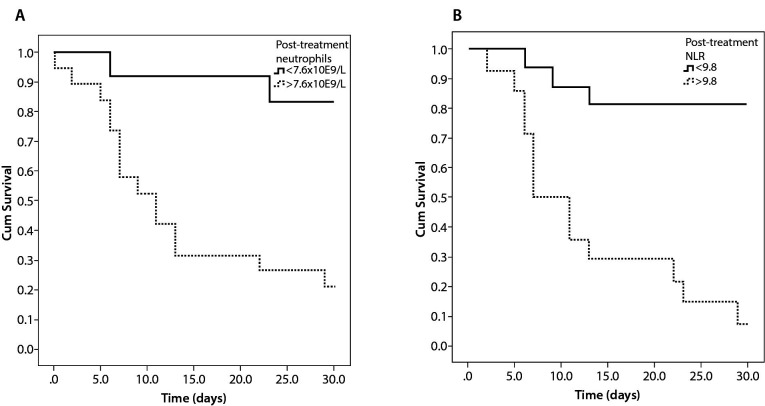
Kaplan-Meier curves showing the difference in survival based on pre-treatment neutrophil count (a) and post-treatment NLR (b). NLR – neutrophil to lymphocyte ratio.

When we analysed the role of pre-treatment parameters as prognostic factors, only total leukocyte, neutrophil count and neutrophil to lymphocyte ratio (NLR) had the ability to predict 30-day mortality (Table 2). Neutrophil count had the best prognostic accuracy (AUC 0.81, 95% confidence interval (CI): 0.65-0.96, P = 0.004). A neutrophil count of 7.6 x10^9^/L had 88% sensitivity and 71% specificity in predicting 30-day mortality. Patients with pre-treatment neutrophil count ≥ 7.6 x10^9^/L had a median survival of 11 days, while the median survival in patients with pre-treatment neutrophil count < 7.6 x10^9^/L was not reached (Log-rank Chi-square 10.8, P = 0.001) (Figure 1a).

**Table 2:** ROC analysis showing the prognostic accuracy of each parameter in predicting 30-day mortality

	**AUC**	**SE**	**P**	**95% CI**	
Age	0.65	0.10	0.165	0.45	0.84
Tocilizumab dose	0.63	0.10	0.234	0.42	0.83
Gender	0.42	0.10	0.475	0.22	0.63
IL-6	0.64	0.11	0.202	0.43	0.85
D-dimers	0.72	0.11	0.081	0.50	0.94
Ferritin	0.70	0.10	0.070	0.51	0.90
**Pre-treatment**					
CRP	0.51	0.11	0.953	0.30	0.71
Leukocytes	0.79	0.08	0.005	0.63	0.96
Neutrophils	0.81	0.08	0.004	0.65	0.96
Lymphocytes	0.41	0.11	0.393	0.20	0.62
NLR	0.77	0.09	0.010	0.60	0.95
**Post-treatment**					
CRP	0.68	0.10	0.096	0.48	0.88
Leukocytes	0.88	0.06	< 0.001	0.75	1.00
Neutrophils	0.94	0.04	< 0.001	0.86	1.00
Lymphocytes	0.86	0.07	0.001	0.71	1.00
NLR	0.94	0.04	< 0.001	0.86	1.00
AUC – area under the curve. SE – standard error. CI – confidence interval. IL-6 – interleukin 6. CRP – C-reactive protein. NLR – neutrophil to lymphocyte ratio. P < 0.05 was considered statistically significant.					

Among post-treatment parameters, both high neutrophil and low lymphocyte count were associated with increased 30-day mortality (Table 2). Both neutrophil count and NLR had similar prognostic accuracy (Table 2). Post-treatment NLR cut-off of 9.8 had the best prognostic capacity with sensitivity of 81% and specificity of 93%. Patients with NLR ≥ 9.8 had the median survival of 7 (3-10) days *vs.* median survival not reached in patients with NLR < 9.8 (Log-rank Chi-square 17.2, P < 0.001) (Figure 1b).

## Discussion

According to the results of our study, patients with severe COVID-19 pneumonia who showed favourable outcome had significantly lower pre-treatment and post-treatment leukocytes, neutrophils and NLR, and also higher post-treatment lymphocyte count. Best prognostic factors for survival in our group of patients were lower pre-treatment neutrophil count and post-treatment NLR. We didn’t find statistically significant difference in 30-day mortality between genders.

Neutrophil to lymphocyte ratio calculated by dividing absolute neutrophil count with absolute lymphocyte count has been reported as an inflammatory biomarker that can be used as an indicator of systemic inflammation with prognostic value in various diseases, such as pneumonia and sepsis (*12*-*14*). A recent study by Liu *et al.* has showed that higher NLR is independently associated with mortality in hospitalized COVID-19 patients (*15*).

Quin *et al.* have shown that higher serum concentrations of proinflammatory cytokines (tumour necrosis factor alpha, IL-1 and IL-6), chemokines and higher NLR in COVID-19 hospitalized patients correlated with the severity of the disease as well as adverse outcome suggesting a potential role of cytokine storm in disease severity (*16*).

Several hypotheses to explain lymphopenia during severe COVID-19 have been proposed. Some of them include T lymphocyte infection by SARS-CoV-2, T lymphocyte exhaustion and cytokine storm (*9*). Additionally, it is suggested that neutrophils with its suppressive effect on lymphocytes are part of impaired anti-viral inflammatory response leading to T lymphocyte anergy (*9*).

Another very important observation shown in study by Zhang *et al.* is gradually increased lymphocyte count in severe COVID-19 cases and also in critical recovered cases. In contrast, lymphocyte count remained low in patients who died (*17*). Furthermore, 85% of critical cases had lymphopenia and patients with fatal outcome had lower lymphocyte count in contrast to critical recovered cases (*17*).

Non-recovering lymphopenia could be a hallmark of severe immune injury and evidence that immunosuppression is more pronounced in critically ill patients with COVID-19 (*8*, *18*, *19*).

We also confirmed that pre-treatment high neutrophil count was associated with adverse outcome. Previous studies have emphasized an increased neutrophil count and decrease in lymphocyte count in COVID-19 patients (*20*, *21*). Qin *et al.* in their study addressed that critical cases have both higher neutrophil and lower lymphocyte count, thus the NLR seemed to be higher in critically ill patients compared to patients who had mild disease (*16*). A permanently high neutrophil count in peripheral blood could be surrogate marker that indicates the intensity of the inflammatory response leading to tissue damage, dominantly in lungs. Maybe these two parameters (higher neutrophil count and NLR), which were proved to be laboratory indicators of severe COVID-19 due to cytokine storm leading to worse outcomes, could be prognostic factors for clinical response to treatment with tocilizumab in patients with high IL-6 concentration. Although our results were similar to previously published studies, we must emphasize the post-treatment neutrophil count, which has the sensitivity 81% and specificity of 93% for adverse outcome. Results of our study showed that patients with severe COVID-19 pneumonia who had high pre-treatment neutrophil count have less chance to respond to tocilizumab as well as higher mortality rate. Therefore, these results implicate that tocilizumab as immunomodulatory therapy alone probably is not sufficient for treating patients with severe COVID-19 pneumonia and high IL-6 concentration who also initially present with high neutrophil count. Furthermore, once who got tocilizumab and have higher post-treatment NLR probably should get additional anti-inflammatory treatment because their response to tocilizumab alone may not be successful (*22*).

This study has several limitations. First, the limited number of patients observed and lack of a randomized control group, which prevents us from deriving definitive conclusions. Second, we also did not measure post-treatment IL-6 concentration to draw more information. Despite this, results shown in this study are in correlation with the previously demonstrated prognostic ability of a high NLR in patients with COVID-19.

In conclusion, patients who survived severe COVID-19 pneumonia treated with tocilizumab had lower pre-treatment and post-treatment neutrophils and lower post-treatment NLR. IL-6 concentrations were not significantly different among patients who recovered and patients with fatal outcomes. Pre-treatment neutrophil count had the best prognostic accuracy in predicting 30-day mortality. Among post-treatment parameters, both neutrophil count and NLR emerged as equally good prognostic factor. Hence, pre-treatment neutrophil count and post-treatment neutrophil count and NLR may help clinicians to identify patients with poor prognosis using a feasible, widely available and inexpensive quantitative tool as a prognostic indicator for adverse outcomes in patients with severe COVID-19 pneumonia treated with tocilizumab and possibly identify once who need additional therapy.
