# Corrigendum to: Post-treatment neutrophil to lymphocyte ratio as a prognostic tool in patients treated with tocilizumab for severe COVID-19 pneumonia - a single center experience

**DOI:** 10.11613/BM.2023.031201

**Published:** 2023-10-15

**Authors:** Marija Gomerčić Palčić, Hana Matijaca, Ivan Kruljac, Lucija Vusić, Vedran Hostić, Luka Vrbanić, Fanika Mrsić, Radovan Zrilić, Ivana Ćelap, Petar Gaćina

**Affiliations:** 1 School of Medicine, University of Zagreb, Zagreb, Croatia; 2 Department of Internal Medicine, Sestre milosrdnice University Hospital Center, Division of Pulmonology, Zagreb, Croatia; 3 Department of Internal Medicine, Sestre milosrdnice University Hospital Center, Division of Hematology, Zagreb, Croatia; 4 Solmed Group, Department: Poliklinika Solmed, Zagreb, Croatia; 5 Department of Anesthesiology, Intensive Care Medicine and Pain Management, Sestre milosrdnice University Hospital Center, Zagreb, Croatia; 6 Department of Internal Medicine, Sestre milosrdnice University Hospital Center, Division of Clinical Immunology and Rheumatology, Zagreb, Croatia; 7 Polyclinic for Respiratory Diseases, Dom zdravlja Zagreb - Zapad, Zagreb, Croatia; 8 Department of Clinical Chemistry, Sestre milosrdnice University Hospital Center, Croatia; 9 School of Dental Medicine, University of Zagreb, Zagreb, Croatia

This is a correction of Biochem Med (Zagreb) 2023;33(2):020704. DOI: https://doi.org/10.11613/BM.2023.020704 

Since the publication of the article, the authors have noticed that their first names and surnames in the by-line were listed in reverse. The correct by-line is presented above. We apologize to the authors for any inconvenience caused to the readers.

